# U24 from *Roseolovirus* interacts strongly with Nedd4 WW Domains

**DOI:** 10.1038/srep39776

**Published:** 2017-01-04

**Authors:** Yurou Sang, Rui Zhang, Walter R. P. Scott, A. Louise Creagh, Charles A. Haynes, Suzana K. Straus

**Affiliations:** 1 Department of Chemistry, The University of British Columbia, Vancouver, British Columbia, Canada; 2 Michael Smith Laboratories and Department of Chemical and Biological Engineering, The University of British Columbia, Vancouver, British Columbia, Canada

## Abstract

U24 is a protein found in both roseoloviruses Human Herpes Virus type 6 and 7 (HHV-6 and HHV-7), with an N-terminus that is rich in prolines (PY motif in both HHV-6A and 7; PxxP motif in HHV-6A). Previous work has shown that the interaction between U24 and WW domains is important for endocytic recycling of T-cell receptors, but a cognate ligand was never identified. In this contribution, data was obtained from pull-downs, ITC, NMR and molecular dynamics simulations to show that a specific interaction exists between U24 and Nedd4 WW domains. ITC experiments were also carried out for U24 from HHV-6A phosphorylated at Thr6 (pU24-6A) and a peptide containing the PY motif from Nogo-A, a protein implicated in both the initial inflammatory and the neurodegenerative phases of multiple sclerosis (MS). The results suggest that phosphorylation of U24 from HHV-6A may be crucial for its potential role in MS.

*Roseoloviruses*, which include human herpes virus type 6A (HHV-6A), HHV-6B and HHV-7, are ubiquitous and highly prevalent. They have been found to be implicated in several neurological diseases, including encephalitis, epilepsy, and multiple sclerosis (MS)^1^,^2^,^3^,^4^. MS is a neurological inflammatory disease in the central nervous system (CNS). The exact cause of MS is unknown, but it has been suggested that one or more viruses may be possible triggers^2^,^5^,^6^. U24 is a putative tail-anchored membrane protein that is unique to roseoloviruses^7^. It is expressed in infected cells during the early stages of viral infection^8^. Although the exact function of U24 has not been identified, a number of studies have demonstrated a potential link between U24 and MS^9^,^10^.

One possible way in which U24 may be implicated in MS is through the mimicry of myelin basic protein (MBP) because the two proteins share a seven amino acid stretch that contains a PxxP motif (Fig. 1a). This segment is essential for MBP to participate in Fyn-mediated signalling pathways by a direct but non-canonical association with the Fyn-SH3 domain^11^. Disruption of these signalling pathways has a large impact on oligodendrocyte differentiation and maturation, processes that are perturbed in MS patients. Within this PxxP motif and just before it are two threonine residues (T95 and T98), which can be phosphorylated. Phosphorylation has been shown to affect the local structure of MBP and its disposition on the membrane surface^12^. U24-6A shares with MBP both the PxxP motif and a threonine at an analogous position to T98 (Fig. 1a). Recent work has demonstrated that U24-6A can be phosphorylated by MAPK, albeit less effectively than MBP^13^. It has also been shown that U24-6A can associate with Fyn SH3, but again does so more weakly than MBP (*K*_*D*_ = 5 mM for U24-6A^14^ versus 4-8 μM for MBP^15^). These findings suggested that although U24-6A may mimic MBP, it most likely does not interfere directly with MBP function.

Within the proline rich region of U24 is a PY motif that is conserved in both HHV-6A and HHV-7 (Fig. 1a). This motif was found to be responsible for the down-regulation of the TCR/CD3 complex in T-cells^16^,^17^. Indeed, instead of being internalized, the TCR/CD3ε receptors were blocked from recycling back to the cell surface^16^. However, the exact mechanism by which this occurs is unknown. Little to no colocalization of U24 and TCR/CD3ε was observed^17^, suggesting that U24, especially its PY motif, may be targeting a process rather than a specific protein. Indeed, U24 was found to also down-modulate the transferrin receptor (TfR)^17^. During the development and maturation of neurons or myelin, many processes need to be tightly regulated. For example, the two minor membrane glycoproteins myelin oligodendrocyte glycoprotein (MOG) and myelin-associated glycoprotein (MAG), both important components of the myelin sheath, have been shown to be endocytosed via a clathrin-dependent pathway^18^. Their fates, however, are different: MAG is targeted to the late endosome and lysosome directly, while MOG is trafficked to the recycling endosome. Another important protein implicated in oligodendrocyte differentiation and myelination is neurite outgrowth inhibitor, Nogo-A, which also has a PY motif (Fig. 1a). Recent work has shown that in MS brain tissue, the expression of Nogo-A was found to be up-regulated at the edge of lesions in oligodendrocytes, suggesting that it may be implicated in demyelination^19^,^20^,^21^,^22^. However, evidence also points to the fact that Nogo-A can have a positive impact on myelination^22^,^23^. In any case, Nogo-A expression is also tightly regulated by Praja2, a member of the RING family of E3 ubiquitin ligases^24^. Finally, sodium channels have been found to be more frequently associated with MS lesions^25^,^26^,^27^. Sodium channels, such as epithelial sodium channel (ENaC), undergo endocytosis through a clathrin-dependent pathway, which is regulated by Nedd4 family E3 ubiquitin ligase^28^. U24’s ability to block endosomal recycling may play a role in perturbing the levels of these proteins, leading to neural tissue damage. In order to lend credence to this hypothesis, a specific interaction between U24 and E3 ubiquitin ligases, e.g. through their WW domains, should be identified. Of all possible ubiquitin ligases to investigate, neural precursor cell (NPC) expressed developmentally down-regulated protein 4 (Nedd4) E3 ubiquitin ligase is an appealing target, as it contains several WW domains and functions in endocytosis and endosomal sorting of membrane proteins^29^,^30^. Specifically in CNS, Nedd4 and Nedd4-like proteins (Nedd4L) play a crucial role in promoting dendrite outgrowth^31^,^32^ and maintaining neuronal survival^33^,^34^. Dysfunction of Nedd4 or Nedd4L may cause defective neural development, resulting in damage of axons or myelin.

Using pull-down assays, Sullivan and Coscoy showed that U24 can bind to most of the seven human Nedd4 family E3 ubiquitin ligases (Nedd4 family E3s) tested: Nedd4-1, Nedd4-2, AIP4, Smurf1, Smurf2, WWP1, and WWP2^35^. However, precise binding affinities are hard to extract from such results. The aim here is to test whether both types of U24, U24-6A and U24 from HHV-7 (U24-7), can bind to Nedd4-WW domains with high affinity and to explain why. We have focussed our studies on human Nedd4 WW domains (hNedd4 and hNedd4L, Fig. 1b) given their relevance in the CNS, noted above. As controls, we have used rat Nedd4 WW domains, which are also canonical WW domains, but have variations in the sequence, in particular in the binding loop (APN in hNedd4 and hNedd4L versus DDR and HTD in rNedd4-WW2 and WW3/4, respectively – Fig. 1b, Supplementary Fig. 1). The binding affinities and location of the binding sites between U24 and all these Nedd4-WW domains were determined using isothermal titration calorimetry (ITC) and heteronuclear single quantum coherence nuclear magnetic resonance spectroscopy (^1^H–^15^N HSQC NMR). Molecular dynamics (MD) simulations were performed to further determine which interactions are key at an atomic level. Finally, some of the data obtained is compared to the binding interaction between the PY motif of Nogo-A and hNedd4L-WW3*, also determined here.

## Results

### Confirmation of protein-protein interactions using GST pull-down assay

In order to compare with previously reported results^35^, experiments were carried out on four relevant WW domains: the second and the third WW domain of rat Nedd4 (rNedd4-WW2 and rNedd4-WW3/4, where WW3/4 is a homologue of the fourth WW domain in human), and the pair of third WW domains from human Nedd4 (hNedd4-WW3* and hNedd4L-WW3*). The WW3 domains are indicated with a star because this type of WW domain is missing in rat and mouse Nedd4^36^,^37^. Nedd4-WW1 domain was not used as it was found previously not to bind U24. The WW domains mentioned above were fused to the C-terminus of GST protein individually. Recombinant full length U24-6A and U24-7 protein were prepared as previously described^14^,^38^.

Bands detected at the U24-6A or U24-7 MWs in lanes 4 and 6 (Fig. 2) indicate that both rat and human Nedd4-WW domains can interact with both U24-6A and U24-7 protein in accordance with the findings of Sullivan and Coscoy^35^.

### Binding affinities between U24 and Nedd4-WW domains

In order to compare the affinities between the U24 proteins and WW domains, the equilibrium dissociation constant (*K*_*D*_) for the interaction between each U24:Nedd4-WW domain pair tested above was determined using ITC. 15-mer peptides, representing the N-terminus of U24-6A and U24-7, were used. ITC experiments using these peptides as titrants were carried out on Nedd4-WW domains in 10 mM sodium phosphate, pH 7.4, at 25 °C. Control experiments in which the phosphate buffer was titrated into the protein solutions were also conducted to ensure the proteins were stable during the titration with peptide.

Overall, the *K*_*D*_ of U24 binding to each Nedd4-WW domain is in the range of 1–150 μM, (Table 1) similar to affinities reported in the literature for other PY motif-containing proteins interacting with WW domains^29^,^36^,^39^. The affinities between U24-7 and the WW3* domains are higher than between U24-7 and the other two ligands or between other U24:ligand pairs. From the perspective of the WW domains, hNedd4-WW3* and hNedd4L-WW3* bind all forms of U24 better than do the other two domains. This is not surprising as this WW3* was found to be the predominant mediator of interactions with PY motif-containing ligands^40^. In the case of rat Nedd4, WW3/4 binds U24 better than does the WW2 domain, similar to the trend observed before^41^. Finally, U24-7 binds WW domains better and has more negative enthalpies than U24-6A peptide (Fig. 3). Since the PY motifs are identical in two U24 peptides, these latter results suggest the residues up- or down-stream from the PY motif may be critical for strong binding.

### Impact of phosphorylation and electrostatics in the interactions

**Figure 1:**
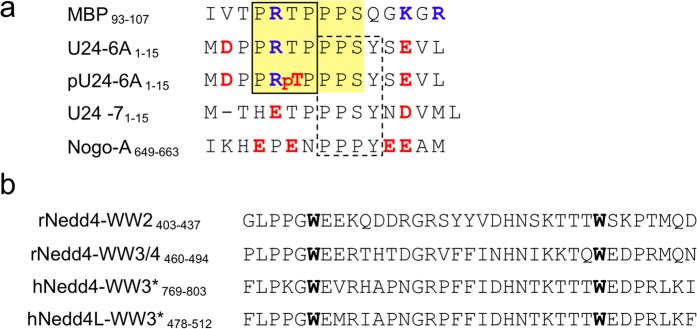
Alignment of (**a**) the proline-rich segments in U24, MBP, and Nogo-A, and (**b**) the WW domains used in this study. (**a**) The numbering of MBP is based on the human 18.5 kDa classic MBP (Note that in ref. 14, the murine numbering 90–104 was used). The identical segment between U24-6A and MBP, PRTPPPS, is marked with yellow shading. The PxxP motif is marked by the solid lined box. U24-7, pU24-6A and Nogo-A are aligned with respect to U24-6A and their common PY motifs are marked with a dashed box. The phospho-threonine in pU24-6A is marked as pT. The positively and negatively charged residues are shown in blue and red, respectively. (**b**) The alignment of WW domains from rNedd4, hNedd4 and hNedd4L. The conserved tryptophans are highlighted in bold. The numbering of the domains is given in the subscript next to the name.

**Figure 2:**
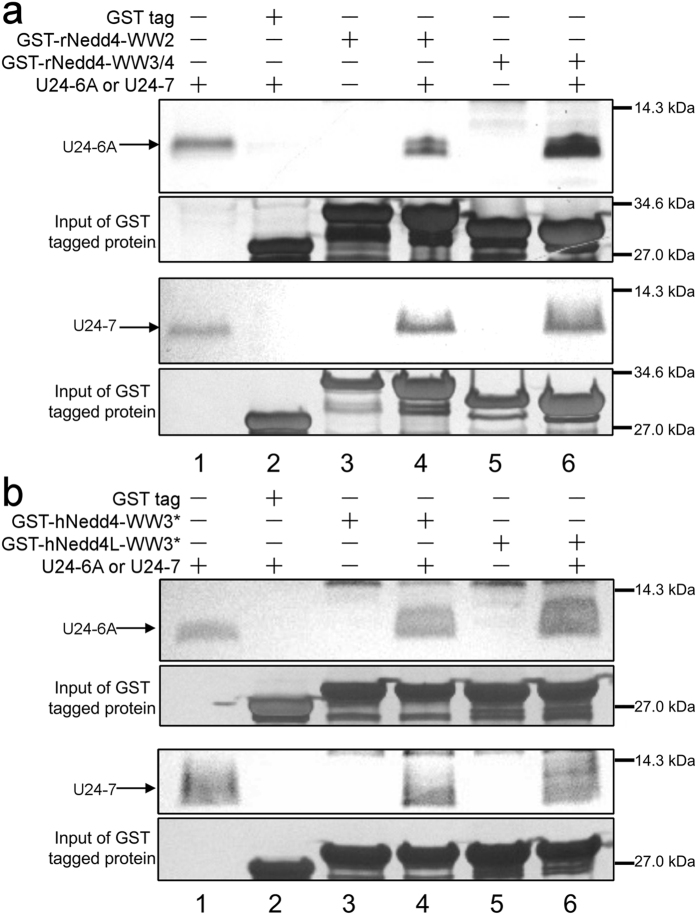
SDS-PAGE result of GST pull-down experiment with GST-Nedd4-WW domains and U24. Pull-down experiment using (**a**) GST-rNedd4-WW2 and GST-rNedd4-WW3/4, (**b**) GST-hNedd4-WW3* and GST-hNedd4L-WW3* and U24-6A and U24-7 protein. U24-6A and U24-7 are loaded in the first lane for reference, and the GST tag alone plus U24 are in the second lane. Lanes 3 and 5 are loaded with GST-Nedd4-WW domains alone. GST-Nedd4-WW domains plus U24 are loaded in lanes 4 and 6. In each case, the bottom gel shows that the amount of GST-tagged protein used is uniform for each combination tested. An uncropped version of this figure can be found in Supplemental Material, Fig. 6.

**Figure 3:**
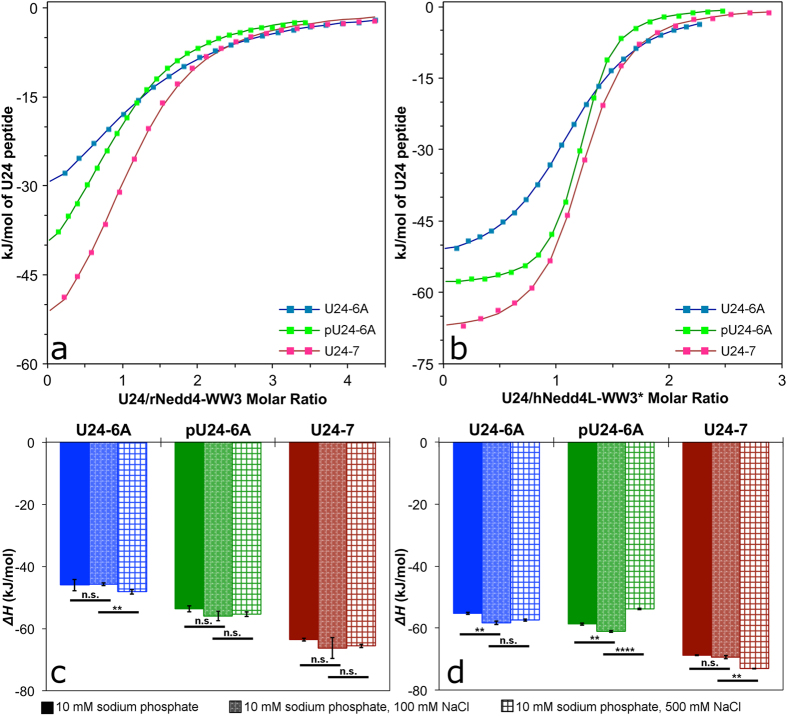
ITC result of three U24 peptide titrated into Nedd4-WW domains. Normalized heats and fits are shown for U24-6A peptide in blue, pU24-6A in green and U24-7 peptide in red. The peptides are titrated into a solutions of (**a**) rNedd4-WW3/4 domain, (**b**) hNedd4L-WW3* domain. The fitted ΔH of ITC experiments done in different buffers were plotted as bar graphs in (**c**) U24 peptides were titrated into rNedd4-WW3/4 domain or (**d**) titrated into hNedd4L-WW3* domain. All titrations are done at 25 °C. Error bars indicate ± one standard deviation, obtained from three repeats. n.s., nonsignificant; *P ≤ 0.037, **P ≤ 0.0088, ***P ≤ 0.0001 by Student’s t test.

As it has been previously demonstrated that U24-6A is extensively post-translationally modified *in vivo*^17^, it is possible that these modifications could play a role in the specific interactions of U24-6A with WW domains. Hence, an additional 15-mer peptide containing a confirmed phosphoryl threonine at Thr6 (pU24-6A) was also studied. ITC experiments were carried out using pU24-6A under the exact same conditions as described above with rNedd4-WW3/4 and hNedd4L-WW3*. The affinities between pU24-6A and the Nedd4-WW domains are better than those between U24-6A or U24-7 and the respective Nedd4-WW domains. In particular, the *K*_*D*_ of pU24-6A binding to hNedd4L-WW3* is 758 ± 27 nM, with the molar enthalpy for pU24-6A binding lying between that of U24-6A and U24-7 (Fig. 3c and d). Importantly, this is the tightest affinity characterized to date between a U24 peptide ligand and WW domains. Finally, for comparison, a peptide consisting of the Nogo-A PY motif (Fig. 1) was tested to determine its affinity to hNedd4L-WW3* and the *K*_*D*_ determined is 4.0 ± 0.2 μM at 25 °C in 10 mM sodium phosphate, pH 7.4 (Table 1).

The factor of three to eight times enhancement in affinities observed for pU24-6A and Nedd4-WW domains may be linked to the charged residue upstream from the PY motif (Fig. 1a). In U24-7, there is a glutamic acid at residue 4; whereas in U24-6A, there is an aspartic acid at position 2, followed by an arginine at residue 5. Phosphorylation at Thr6 would provide an extra negative charge for pU24-6A. There are several conserved lysines or arginines in Nedd4-WW domains (Fig. 1b) that could provide the right counter charge for the pU24-6A and U24-7 ligands, and hence account for the more favourable interactions between these pairs.

In order to find out whether electrostatic interactions play a role in the binding of the three U24 peptides tested here to the Nedd4-WW domains, a set of ITC experiments were performed as a function of increasing salt concentration, 0 mM, 100 mM and 500 mM, in 10 mM sodium phosphate, pH 7.4, at 25 °C, for rNedd4-WW3/4 and hNedd4L-WW3*. Affinities and molar binding enthalpies (ΔH) for each U24:WW-domain pair as a function of salt concentration are plotted in Supplementary Fig. 2 and Fig. 3c and d, respectively.

Little change in ΔH for a given ligand binding to rNedd4-WW3/4 is observed as a function of increasing salt concentration. Considering the error bars shown, no clear trend can be obtained from a comparison of the ΔH values, suggesting that there is no strong electrostatic interactions at play in this protein-ligand interaction. On the other hand, there are statistically significant changes in ΔH for the case of pU24-6A or U24-7 interacting with hNedd4L-WW3* (Fig. 3d). Interestingly, the trends observed are different for the two ligands. ΔH for U24-7 interacting with hNedd4L-WW3* becomes more negative with increasing salt concentration, whereas that of pU24-6A becomes less negative at high salt concentration (500 mM NaCl). This latter trend could result from specific salt bridges between pU24-6A and hNedd4L-WW3* being disturbed by shielding effects from the surrounding ions.

The *K*_*D*_ values of all three U24 ligands binding to rNedd4-WW3/4 decrease with increasing salt concentration (Supplementary Fig. 2a), indicating that the affinities are getting stronger. On the other hand, the *K*_*D*_ plot for hNedd4L-WW3* shows the opposite trend (Supplementary Fig. 2b). The affinities between pU24-6A or U24-7 and hNedd4L-WW3* are found to decrease as the salt concentration increased, while that of U24-6A seems to be unaffected. Moreover, the equilibrium constant of pU24-6A weakens more significantly than that of U24-7 upon salt addition. The *K*_*D*_ values of pU24-6A and U24-7 in 500 mM NaCl are almost the same.

### Residues located at the binding site

**Figure 4:**
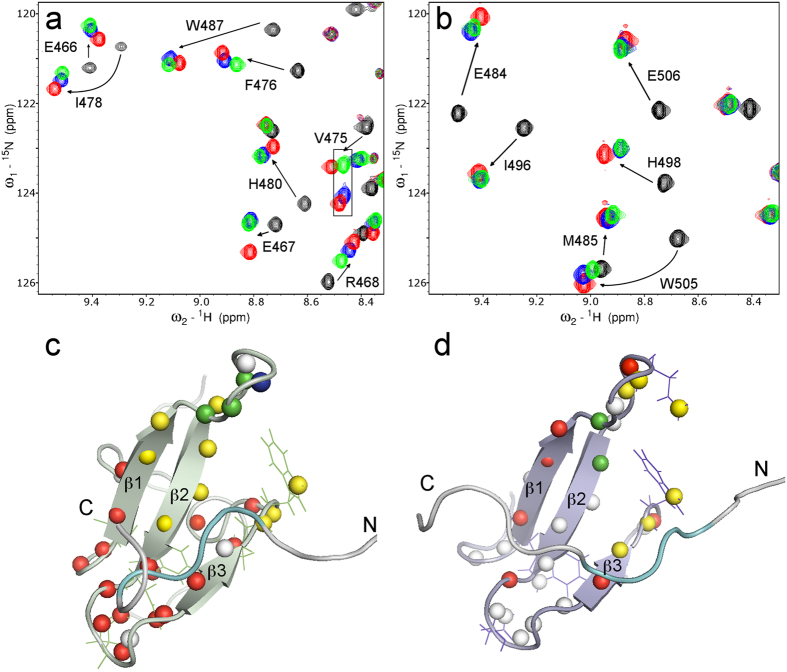
Regions of NMR HSQC spectra overlays and structural models coloured according to which residues are perturbed. NMR HSQC overlays for apo and complexed (**a**) rNedd4-WW3/4 and (**b**) hNedd4L-WW3*. The resonances of apo WW domains are shown in black. WW domain bound with U24-6A are shown in blue; bound with pU24-6A are shown in green; and bound with U24-7 are shown in red. (**c**) Coloured model of rNedd4-WW3/4 and PY motif peptide (PDB: 1I5H). The WW domain is shown in green and peptide is shown as a grey ribbon with PY motif region highlighted in cyan. The nitrogen atoms are shown as spheres and coloured based on which ligand perturb the amide differently from the other two. Blue means U24-6A; red is U24-7; green is pU24-6A; yellow means three ligands perturb this amide very differently; and white means three ligands perturb this amide similarly. (**d**) Coloured model of hNedd4L-WW3* and PY motif peptide (PDB: 2MPT). The WW domain is shown in purple and peptide is shown as a grey ribbon with PY motif region highlighted in cyan. Other colour scheme is as in (**c**).

In addition to determining the affinities between U24 and the Nedd4-WW domains using ITC, ^1^H–^15^N HSQC NMR spectroscopy was performed to characterize the binding site in rNedd4-WW3/4 and hNedd4L-WW3* domains upon ligand binding. ^15^N-labelled rNedd4-WW3/4 and hNedd4L-WW3* were used in the experiment and the spectra of apo WW domain were recorded at 25 °C in 10 mM phosphate buffer, pH 7.4. HSQC spectra of six protein-peptide complexes, involving the three U24 ligands and two WW domains, were also recorded under the same conditions. The assignment of apo rNedd4-WW3/4 and hNedd4L-WW3* domains and the complexes were achieved using 3D HSQC-NOESY and 3D HSQC-TOCSY experiments. The spectra and where binding occurs on the structural models (based on PDB entries 1I5H and 2MPT, respectively) are shown in Fig. 4.

The Δδ_comb_ calculated using the changes in ^1^H and ^15^N chemical shifts were found to be similar for the three peptides (Supplementary Fig. 3). Indeed, the peak positions seen in the overlays of the HSQC spectra in Fig. 4 are generally quite close, with a few exceptions. The residues perturbed the most in rNedd4-WW3/4 and hNedd4L-WW3* were on its third β sheet (β3 sheet), which is important for binding PY motif ligands^41^. The loop between β1 and β2 (β1-β2 loop) was also strongly perturbed in rNedd4-WW3/4, whereas the proline in the β1-β2 loop of hNedd4L-WW3* might limit the flexibility of the loop, resulting in fewer perturbations of the backbone.

In the spectra of rNedd4-WW3/4 and hNedd4L-WW3*, most amide resonances showed similar chemical shift perturbations when complexed with U24-6A and pU24-6A, indicating that the backbone is perturbed similarly by these peptides. Indeed, most of the blue and green peaks in Fig. 4 (Fig. 4a and b) are overlapped. In order to emphasize the differences in binding of the three U24 ligands better, the NMR chemical shifts were analyzed in the following manner: for a given ligand, the peak position was deemed different if the chemical shift changed by 0.15 ppm or more in the ^15^N dimension or 0.02 ppm or more in the ^1^H dimension. Figure 4 shows structural models of the rNedd4-WW3/4 and hNedd4-WW3* domains color-coded according to which residues were deemed different according to the criteria given above (see legend for color codes). U24-7 ligand mainly perturbs the region where the second half of the PY motif peptide (middle residue to C-terminus) binds, while perturbations in the β1-β2 loop are mostly introduced by the pU24-6A ligand. The residues around the conserved tryptophan are perturbed differently among the three ligands. Interestingly, this analysis suggests that the two WW domains show slightly different patterns of U24 peptide binding. In particular, the phospho-ligand affects different residues on the WW domains.

### MD simulations support the role of the N-terminal residues

**Figure 5:**
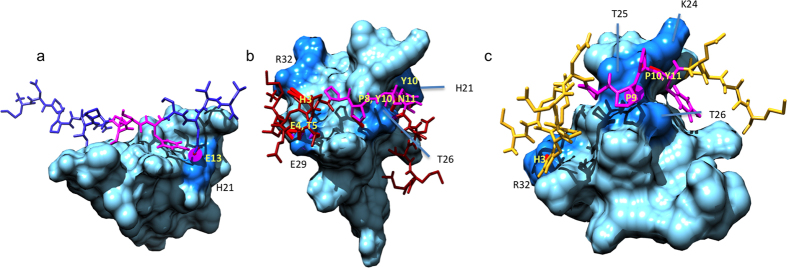
Representative structures observed during the MD simulations of (**a**) hNedd4L-WW3* and U24-6A peptide. (**b**) hNedd4L-WW3* and U24-7 peptide. (**c**) hNedd4L-WW3* and Nogo-A peptide, the sequences of which are given in Fig. 1a. The PY motifs are indicated in magenta. The WW domains are represented as space-filling models, with the darker blue representing the residues involved in contacts with the peptides. The N-terminus is always located to the left. The view of the WW domain is rotated to illustrate as many contacts as possible, indicated in black (hNedd4L-WW3*) and yellow (peptide ligand). For a full list of contacts, see Supplementary Tables 2 and 3.

To further pinpoint the binding interactions, MD simulations were conducted of rNedd4-WW3/4 with U24-6A and U24-7 peptides. Simulations of hNedd4L-WW3* with these same peptides and the segment of Nogo-A given in Fig. 1 were also carried out. All simulations were 30 ns in length and in explicit water. In most cases, close interactions between the PY motif and the WW domains are evident, as seen by looking at the magenta segment of the peptides in Fig. 5 and hNedd4L-WW3* (see also Supplementary Fig. 4). The exception is the simulation involving rNedd4-WW3/4 with U24-6A (Supplementary Fig. 4), where the peptide is not preferentially in close proximity to the WW domain. The representative structures, obtained through a cluster analysis, correlate roughly to the *K*_*D*_ values obtained from the ITC measurements and suggest that while the interaction with the PY motif is key, the interaction with residues upstream from the PY are also very important for stabilizing the binding. Interestingly the number of hydrophobic contacts and hydrogen bonds is largest between U24-7 and hNedd4L-WW3* (Supplementary Table 2), with a key contact between R509 of the WW domain and a histidine at residue 3 in U24-7. This same contact stabilizes the interaction between hNedd4L-WW3* and Nogo-A, which also has a histidine at the third residue in the segment simulated. Another residue that both peptides interact with is residue T503 in hNedd4L-WW3*. Hence, it would appear that it is the residues that are close to the second tryptophan (W505, Supplementary Fig. 3) which are important. In other words, the WW domain binding loop residues (APN in hNedd4 and hNedd4L versus DDR and HTD in rNedd4-WW2 and WW3/4, respectively) are not critical, at least as far as the short peptide segments simulated here suggest.

## Discussion

The results presented show that U24-6A and U24-7 protein, and peptides consisting of the first 15 residues in the sequence, can bind to all four WW domains tested. Interestingly, a phosphorylated version of U24-6A, pU24-6A, is found to have the best affinities with the WW domains. Overall, the dissociation constants between the U24 peptides and the WW domains are of order μM, indicating strong binding. Importantly, the affinities for U24 peptides binding to the WW3* domain of hNedd4 or hNedd4L at 25 °C, especially pU24-6A, are better than most of the ligands reported to date in the literature on single WW3* domains, the dominant mediator of hNedd4 protein^41^,^42^,^43^,^44^. This strong interaction suggests that U24 could compete with or inhibit the intrinsic interactions of WW3* domains, or even hNedd4 protein *in vivo*, though explicit *in vivo* tests are required to prove this. In other words, the strong binding observed and described here may mean that U24 interferes with the function of Nedd4 as a protein regulator. The ramifications of this could, of course, be very complicated and are beyond the scope of this contribution, but the work presented here demonstrates that even though the exact function of U24 is still uncertain, it is highly possible it is closely related to that of Nedd4. In other words, the strong interactions elucidated here suggest that U24 has a specific interaction partner, unlike what was previously suggested^17^,^35^.

The thermodynamic parameters obtained for the U24:WW domain complexes show that these interactions are enthalpy-driven. The interactions with U24-7 were always found to have a more favorable ΔH than those with U24-6A and pU24-6A, suggesting formation of a higher density of favorable bonds, a finding that is supported by the MD results. This observation may be due to differences in the types of residues flanking the identical PY motifs in the three U24 ligands studied. The charged residue or phosphoryl group upstream from the PY motif seems to be able to enhance its affinities with WW domains (Fig. 1), even though an earlier study suggested that only the core PY motif residues is required for high affinity binding^45^,^46^. The charged residue or phosphoryl group are also responsible for the data observed in the electrostatic effect investigations. As for hNedd4L-WW3* domain, the affinities of pU24-6A and U24-7 to it were weakened upon increasing salt concentration, different from all other U24:WW pairs tested. |ΔH| of pU24-6A is significantly reduced at high salt concentrations, suggesting fewer bonds are formed upon binding. The dependence of ln *K*_*D*_ on salt concentration can be used to estimate the total number of cations, anions or water molecules released during the interaction, which for pU24-6A and U24-7 are about −0.2 and −0.1, respectively^47^,^48^,^49^. This indicates that ion release upon binding is negligible, indicating that the contribution of electrostatic interactions to complex stability is relatively minor, consistent with the weak dependence of *K*_*D*_ on ionic strength (Fig. 3c,d). The solvent-exposed binding surface areas on both rNedd4-WW3/4 and hNedd4L-WW3* were also investigated, but no distinct differences were observed (Supplementary Fig. 3). This is again consistent with the MD simulations, which suggest that the tight binding is driven by van der Waals interactions, with hydrophobic contacts and hydrogen bonds providing the dominant contributions.

To put the findings obtained here into the context of a potential link between U24 and MS, it is interesting to compare the binding affinities and structural models of hNedd4L-WW3* interacting with pU24-6A, U24-7, and Nogo-A. It was found that the interaction of hNedd4L-WW3* with Nogo-A is not as strong as with pU24-6A, or with U24-7 peptide, suggesting the two glutamic acids upstream from the PY motif in Nogo-A may not contribute much to the interaction. The question that remains is why? Our collective data show that the interactions stabilizing the pU24-6A:hNedd4L-WW3* complex are not purely electrostatic. The presence of an additional charged residue, as in the Nogo-A peptide, does not improve binding. Moreover, the weak dependence of *K*_*D*_ on ionic strength and the molecular dynamics simulations indicate that binding is primarily driven by hydrophobic contacts and hydrogen bonding. But salt bridges among charged residues must form to satisfy electroneutrality in poorly solvated environments, suggesting that energetically preferred salt-bridge geometries might also contribute to stability^50^. This could help explain the relatively high affinities observed for both pU24-6A and U24-7. Not only does the phosphoryl group elongate the side chain of the threonine, it also provides a large polar interface to interact with the WW domain. Clues to the involvement of the phosphoryl group and flanking residues could be found in the NMR results. The backbone and side chain amides of N490 and R492 are perturbed differently by pU24-6A peptide. These two residues could potentially form complex hydrogen bonds/salt bridges with pU24-6A. They are in the same region and near the binding site of the PY motif, the second conserved W505 in the WW domain. Indeed, both N490 and R492 have been identified as a binding site for phospho-serine in a triphosphorylated Smad3 peptide^39^. The dissociation constant between triphosphorylated Smad3 and hNedd4L-WW3* is in the range of high nanomolar^39^, similar to that of pU24-6A. This strongly suggests that these two residues could be key to the phospho-ligand preference of hNedd4L-WW3*. Even though it is a very weak electrostatic interaction, an enhancement in affinities could be observed with pU24-6A, when compared to U24-6A. In the case of U24-7, there is a glutamic acid upstream from the PY motif, which might have a similar role as the phosphoryl group. This negative charge is closer to the backbone of the peptide, so the U24-7 peptide might be brought closer to WW domains than other ligands. This could be explained as the special perturbation of U24-7 in the second half of binding sites in WW domains. Overall, the phosphorylation or negatively charged residue upstream from the PY motif can enhance the affinities with hNedd4L-WW3* domain.

In summary, we have demonstrated that a cognate ligand of U24 exists, namely hNedd4L-WW3* domain. In addition, the interaction was found to be slightly stronger than that between hNedd4L-WW3* domain and Nogo-A, suggesting that U24 could potentially play a role in interfering with Nogo-A levels. Further *in vivo* testing would be needed to support this suggestion. An “interference” by U24 could have a direct impact by influencing Nogo-A’s function of myelination^22^,^23^. Alternatively, U24 may mimic Nogo-A in lesions in oligodendrocytes, suggesting that it too may be implicated in demyelination^19^,^20^,^21^,^22^. Of course, U24 may function in an entirely different manner, which does not involve Nogo-A. What we have demonstrated here, however, is that E3 ubiqitin ligases are likely to be tightly linked to U24. Overall, our findings suggest that U24 could be a potential key player in MS or other demyelinating diseases and should be investigated further.

## Methods

### Cloning, expression and purification of WW domains

Two cDNA clones containing human E3 ubiquitin-protein ligase Nedd4 (KIAA0093) and Nedd4-like protein (KIAA0439) were ordered from Kazusa DNA research institute (Chiba, Japan). The DNA fragment of the third WW domain of both proteins was amplified and inserted into BamHI and XhoI sites of pGEX-4T2 vector (GE Healthcare). The plasmids containing GST fusion proteins of the second and third WW domains (WW2 and WW3/4) of rNedd4 were kind gifts from Dr. Julie D. Forman-Kay (Hospital for Sick Children, Toronto). All plasmids were transformed into *E. coli* BL21(DE3) for expression at 25 °C for 16 hours using a final concentration of 400 μM IPTG. ^15^N labelled protein was expressed in M9 media supplemented with ^15^N labelled NH_4_Cl. The cells were harvested by centrifugation and stored in the −80 °C freezer until further use. The cell paste from approximately 600 mL culture was thawed on ice and resuspended in 10 mL PBS supplemented with 1% Triton X-100 (PBS/Triton buffer). Lysozyme, protease inhibitor and DNAse were added in the mixture. The mixture was incubated on ice for half an hour, then was lysed in an ice bath by sonication. After being centrifuged at 9,000 rpm for 1 hour, the supernatant was filtered using a 0.45 μm filter, and then applied to 2 mL of PBS washed Glutathione Sepharose 4B resin (GE Healthcare, GST 4B resin). The resin slurry with cell lysate was incubated for 2 hours at room temperature, and then washed with PBS/Triton buffer six times. The resin was collected by centrifugation at 600 × *g* for 5 minutes.

The GST tagged WW domains were eluted using GST elution buffer (50 mM Tris-HCl, 10 mM reduced glutathione, 1% Triton X-100, pH 8.0). 3 mL of GST elution buffer was used to wash the resin two more times. The eluted fusion protein was dialyzed against 2 L of pull down buffer (20 mM KH_2_PO_4_, 75 mM NaCl, 0.5% Triton X-100, pH 7.4) twice overnight. After the dialysis, the GST fusion WW domain was filtered using a 0.45 μm filter and its concentration was determined by BCA protein assay (Pierce).

In order to obtain WW domains without the GST tag, the proteins were extracted and immobilized on GST 4B resin as described above. The resin was washed three times with PBS buffer, after the washing with PBS/Triton buffer. 3 mL PBS buffer and 150 units of thrombin (GE Healthcare) were added into the pelleted resin and the slurry was incubated at room temperature for 16 hours. The supernatant containing cleaved WW domains were collected on the second day by centrifugation. The residual thrombin in this fraction was removed using p-Aminobenzamidine-agarose (Sigma-Aldrich) and fresh GST 4B resin was used to remove the GST contaminants. The WW domain containing fraction was dialyzed at 4 °C against 2 L phosphate buffer (10 mM sodium phosphate, pH 7.4) using 2,000 MWCO dialysis membrane overnight for ITC or NMR experiments, or phosphate buffer with various salt concentrations (phosphate buffer with 100 mM or 500 mM NaCl) for ITC experiments.

### GST tagged WW domains pull-down assays with recombinant U24-6A and U24-7 protein

The GST-Nedd4 WW domains were prepared as described above, and approximately 1 to 2 nmole of protein was immobilized on 20 μL GST 4B resin individually. U24-6A and U24-7 were prepared as described previously^14^. The resin was incubated with 600 μL U24-6A and U24-7 protein stock solution in pull down buffer at 4 °C. U24-7 stock solution was supplemented with 1 mM DTT. After 1 hour of incubation, the resin was washed three times in order to remove the free U24 protein from the system. Before being mixed with gel loading buffer, the resin was washed using Glasgow lysis buffer for a last time. The samples were heated at 95 °C for 5 minutes and then were centrifuged. 3 to 10 μL of the supernatant was removed, and loaded onto a 0.75mm SDS-PAGE gel that was run using a Tris-Tricine buffer system. The gels were stained with silver stain according to standard protocols.

### Isothermal Titration Calorimetry analysis

After overnight dialysis, the cleaved Nedd4 WW domains in phosphate buffer, with different salt concentrations, were ready for use in ITC experiments. The dialysis buffer was kept as a sample-free buffer to measure the heats of dilution. The sample was then concentrated to 40 to 150 μM, depending on the estimated *K*_*D*_ value, using Microsep centrifugal devices with a MWCO of 1 kDa. The concentration of the sample was determined by the absorbance at 280 nm in 6 M Guanidine HCl on a UV-Vis spectrophotometer, and calculated using the theoretical extinction coefficient obtained from the ProtParam tool^51^. A 15× to 30× concentrated peptide stock solution was made by dissolving the purified peptides, U24-6A, pU24-6A and U24-7, in the same buffer as the protein and its pH was adjusted to match the buffer within 0.05. The peptide amounts were determined gravimetrically and calibrated according to the absorbance at 280 nm in 6 M Guanidine HCl. Both protein and peptide stock solutions were filtered and degassed before loading into the sample cell and injection syringe, respectively.

ITC experiments were performed on a MicroCal VP-ITC (Malvern Instruments) at 15 °C, 25 °C and 37 °C. The titration protocol was comprised of a preliminary injection of 2 μL of the peptide solution, followed by 20 or 25 consecutive 10 μL injections into the sample cell (1.4 mL) containing the Nedd4-WW domain studied. The time between each injection was 300 seconds. Control titrations of peptide solution into protein-free buffers were also conducted at different temperatures. The heats of dilution for the peptide were subtracted from the original heats prior to data fitting to a bimolecular interaction model to obtain *n, K*_*D*_ and ∆H. The ITC experiments were repeated three times with mean values and standard deviations reported. The experiments of buffer titrated into a Nedd4-WW domain were carried out at different conditions to monitor the state of the protein during titration, and a small aliquot of sample taken before and after titration was analyzed on SDS-PAGE to confirm there was no degradation.

### ^1^H-^15^N NMR experiments of WW domains

NMR experiments were conducted on ^15^N labelled rNedd4-WW3/4 and hNedd4L-WW3* domains. Both domains were handled in the same manner, so they are denoted as WW3 domain in this paragraph for simplification. Starting from purified protein, as described above, ^15^N labelled WW3 domain protein was prepared by overnight dialysis against 10 mM sodium phosphate, pH 7.4. The protein sample was concentrated using a Microsep centrifugal device with a MWCO of 1 kDa. Protein solutions were centrifuged several times until a final concentration of about 0.4 to 0.6 mM in 10 mM sodium phosphate, pH 7.4 was reached. The sample was then supplemented with 10% D_2_O, 0.5 mM benzamidine, 0.1% sodium azide. The concentration determination and buffer matching of the WW3 domains and three peptide stock solutions was conducted as described above.

NMR experiments on rNedd4-WW3/4 and hNedd4L-WW3* were performed at 25 °C using a Bruker Avance III 850 MHz NMR spectrometer (Milton, Ontario, Canada), equipped with a TCI probe. ^1^H–^15^N HSQC spectra of apo protein, in a 5 mm NMR tube, were recorded. Then, the U24 peptide stock solution, normally 3–8 mM, was added into the NMR sample in small aliquots and additions were stopped when there were no additional chemical shift changes observed. The ^1^H–^15^N HSQC of six U24/WW domain complexes, three U24 ligands with rNedd4-WW3/4 and hNedd4L-WW3*, were recorded using the same program as used above for the apo protein. The data sets were processed using NMRpipe^52^, and visualized using Sparky^53^. The resulting spectra of apo and bound protein were assigned based on published assignments (BMRB entries 4963 and 25000 for rNedd4-WW3/4 and hNedd4L-WW3*, respectively) and verified using additional 3D HSQC-NOESY and 3D HSQC-TOCSY spectra. The chemical shift changes of the amide protons, Δδ_HN_ in the proton dimension and Δδ_N_ in the nitrogen dimension, were calculated according to Equation (1). The Δδ_comb_ was calculated (using Equations (2) and (3))^54^,^55^ for each residue to represent the chemical shift perturbations upon U24 ligand binding.


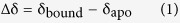



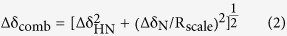



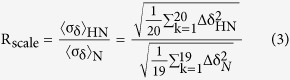


### Molecular Dynamics Simulations

Four simulations were set up using the starting structure deposited in the Protein Data Bank (PDB) (entry 2MPT, model 1). The simulations were run on 2MPT (WW3 domain of Nedd4L in complex with its HECT domain PY motif) for comparison with an experimental structural model. In the other three simulations, the coordinates of the U24 and Nogo-A peptides investigated (Fig. 1) were generated by using the “swapaa” functionality in UCSF CHIMERA^56^. The GROMOS96^57^ biomolecular simulation package and the 53A6^58^ force field were used. The models were solvated in explicit SPC water^59^. Rectangular periodic boundary conditions were imposed. Simulations were performed in the NPT ensemble (T = 300 K, and P = 1 atm) using the Berendsen weak coupling methods^60^. Covalent bonds were constrained using the SHAKE method, with a relative geometric tolerance of 10^−4^ 61. A reaction field long-range correction to the truncated Coulomb potential was applied^62^. Each of the simulations was run for 30 ns, after equilibration for 1 ns.

Three hundred configurations equidistant in time were selected from each 30 ns trajectory and subjected to further analysis. An analysis of hydrogen bond frequencies and hydrophobic contacts, as well as a cluster analysis, as previous described^63^, were performed. Clustering partitions conformations visited during the course of a simulation into subsets called clusters by geometric similarity regardless of the evolution of time. The algorithm determines a representative structure of each cluster. Here, we select the representative structure of the largest determined cluster of a given simulation for visualization purposes.

## Additional Information

**How to cite this article**: Sang, Y. *et al*. U24 from *Roseolovirus* interacts strongly with Nedd4 WW Domains. *Sci. Rep.* **7**, 39776; doi: 10.1038/srep39776 (2017).

**Publisher's note:** Springer Nature remains neutral with regard to jurisdictional claims in published maps and institutional affiliations.

## Supplementary Material

Supplemental Material

## Figures and Tables

**Table 1:** Parameters obtained from fitting the ITC data for binding of U24 peptides to Nedd4-WW domains at 25 °C.

	U24-6A		pU24-6A		U24-7		Nogo-A	
	*K*_D_ (μM)	ΔH (kJ/mol)	*K*_D_ (μM)	ΔH (kJ/mol)	*K*_D_ (μM)	ΔH (kJ/mol)	*K*_D_ (μM)	ΔH (kJ/mol)
rNedd4-WW2	155 ± 12	−54 ± 5			87 ± 5	−76 ± 3		
rNedd4-WW3/4	43 ± 2	−46 ± 2	13.5 ± 0.4	−54 ± 1	21.0 ± 0.5	−63.5 ± 0.5		
hNedd4-WW3*	9.7 ± 0.2	−53.0 ± 0.1			1.98 ± 0.04	−70.1 ± 0.2		
hNedd4L-WW3*	6.3 ± 0.3	−55.3 ± 0.3	0.76 ± 0.03	−58.7 ± 0.4	1.22 ± 0.01	−68.8 ± 0.1	4.0 ± 0.2	−57.9 ± 0.9

The parameters are obtained from fitting the data from three separate runs and averaging them. The errors represent ± one standard deviation.
