# Supplementary material for: U24 from Roseolovirus interacts strongly with Nedd4 WW Domains
Source: Sci Rep. 2017 Jan 4;7:39776. doi: 10.1038/srep39776 (PMC5209733; doi:10.1038/srep39776)
Supplement: Supplemental Material [file srep39776-s1.pdf]

Supplementary Material to

U24 from *Roseolovirus* interacts strongly with Nedd4 WW Domains

Yurou Sang[1], Rui Zhang[1], Walter R P Scott[1], A Louise Creagh[2], Charles A Haynes[2], Suzana K Straus[1]*

[1] Department of Chemistry, The University of British Columbia, Vancouver, British Columbia, Canada.

[2] Michael Smith Laboratories and Department of Chemical and Biological Engineering, The University of British Columbia, Vancouver, British Columbia, Canada.

* e-mail: sstraus@chem.ubc.ca

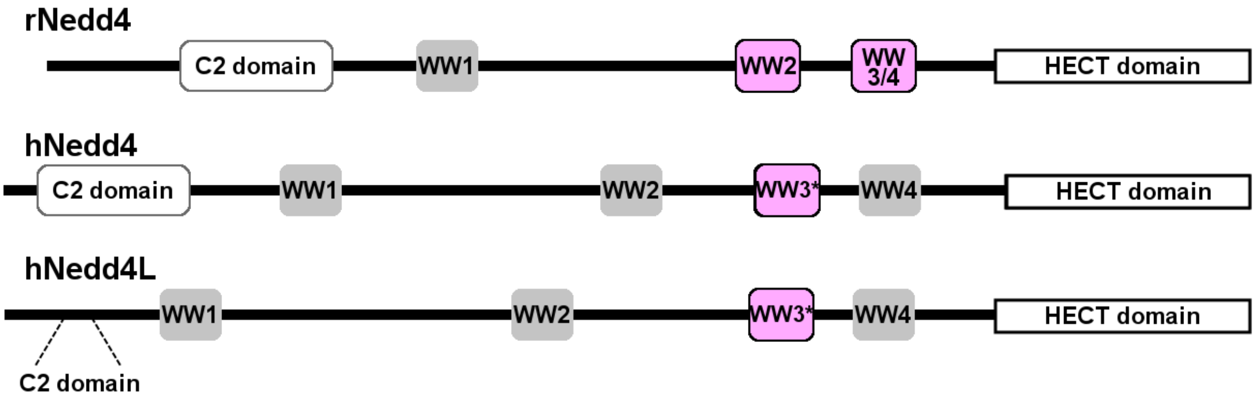

Supplementary Figure 1 Schematic architectures of Nedd4 and Nedd4L protein.

The WW domains used in this chapter are highlighted in pink in the figure above. The dashed line and C2 domain in hNedd4L indicates some hNedd4L isoforms do not have a C2 domain.

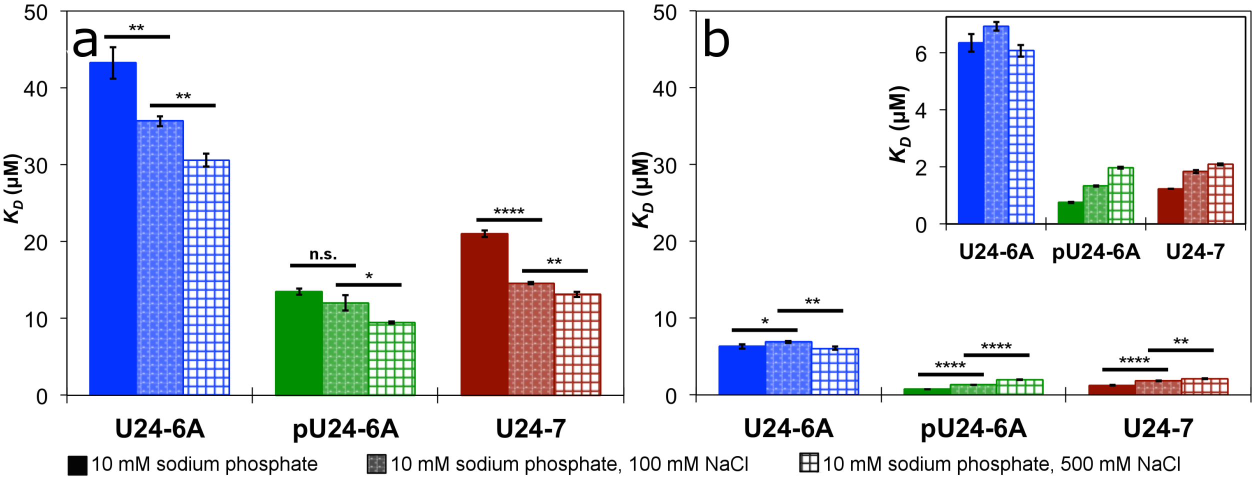

Supplementary Figure 2 K_D plots of the three U24 ligands binding to Nedd4-WW domains in phosphate buffer, phosphate buffer with additional 100 mM NaCl or 500 mM NaCl at 25 °C.

K_D plot of the three ligands titrated into a) rNedd4-WW3/4 or b) hNedd4L-WW3* in three different buffers. Solid bars indicate that the experiments were done in 10 mM sodium phosphate, pH 7.4. Bars with a dotted pattern were for experiments done in phosphate plus an extra 100 mM NaCl, while bars filled with a grid pattern were for experiments where an extra 500 mM NaCl was added to the phosphate buffer. Error bars indicate ± one standard deviation, obtained from three repeats.

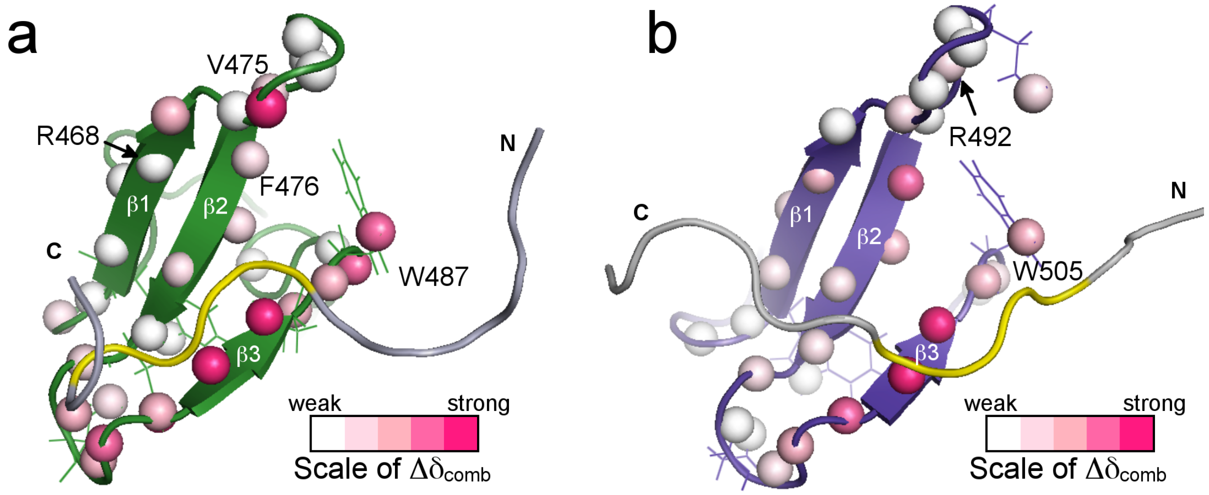

Supplementary Figure 3 Combined chemical shift perturbations mapped on a) rNedd4-WW3/4 and b) hNedd4L-WW3* domain when complexed with U24 peptides.

a) Coloured model of rNedd4-WW3/4 and a PY motif peptide (PDB: 1I5H). The WW domain is shown in green and the peptide is shown as a grey ribbon. The PY motif is highlighted in yellow. The nitrogens in amides are shown as spheres and coloured according to the $\Delta\delta_{comb}$ scale described methods. b) Coloured model of hNedd4L-WW3* and a PY motif peptide (PDB: 2MPT). The WW domain is shown in purple. The $\Delta\delta_{comb}$ data was mapped onto this domain as described in a).

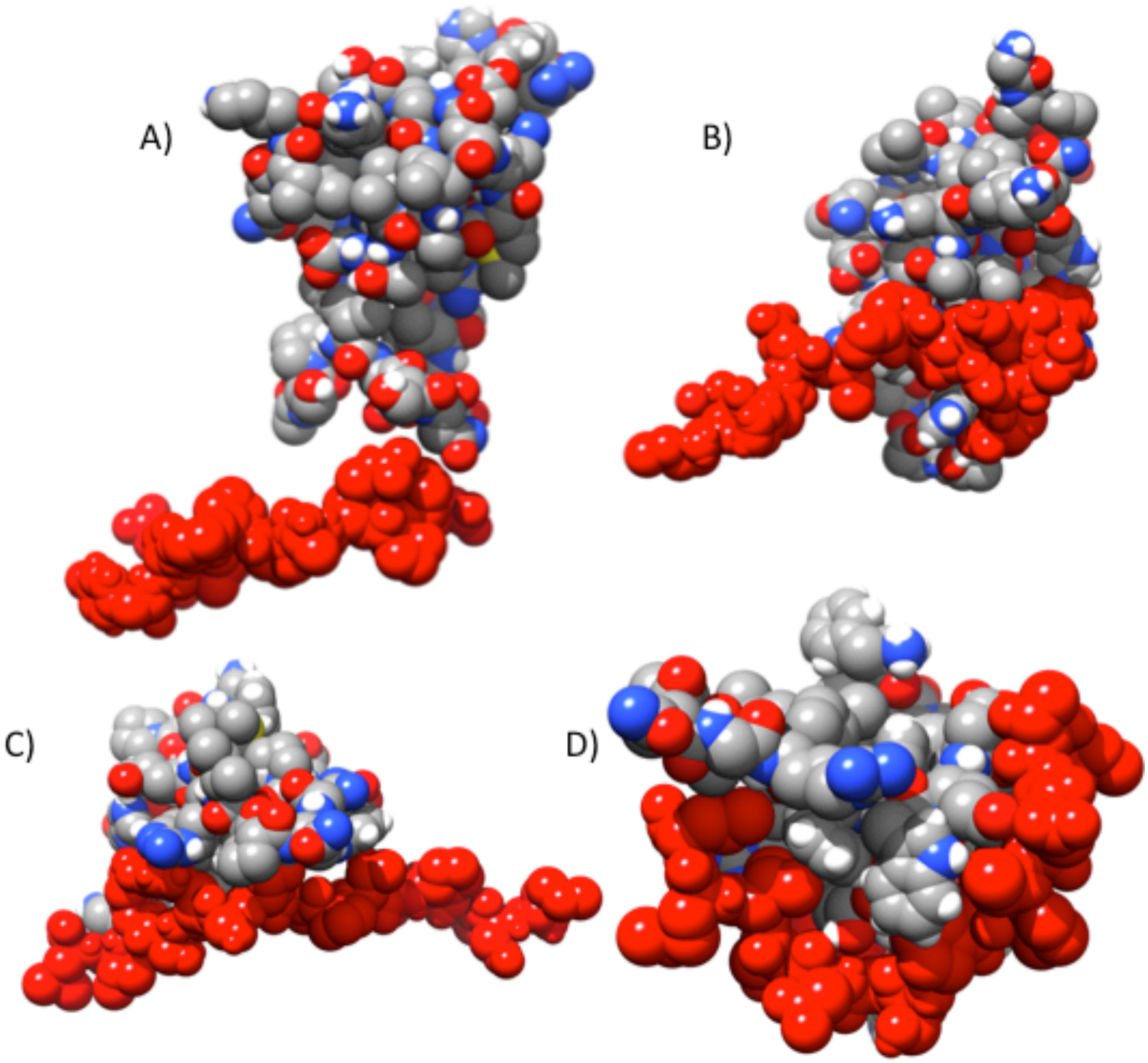

Supplementary Figure 4 Representative structures from MD simulations of rNedd4-WW3/4 with a) U24-6A and b) U24-7 peptides; and hNedd4L-WW3* with c) U24-6A and d) U24-7 peptides.

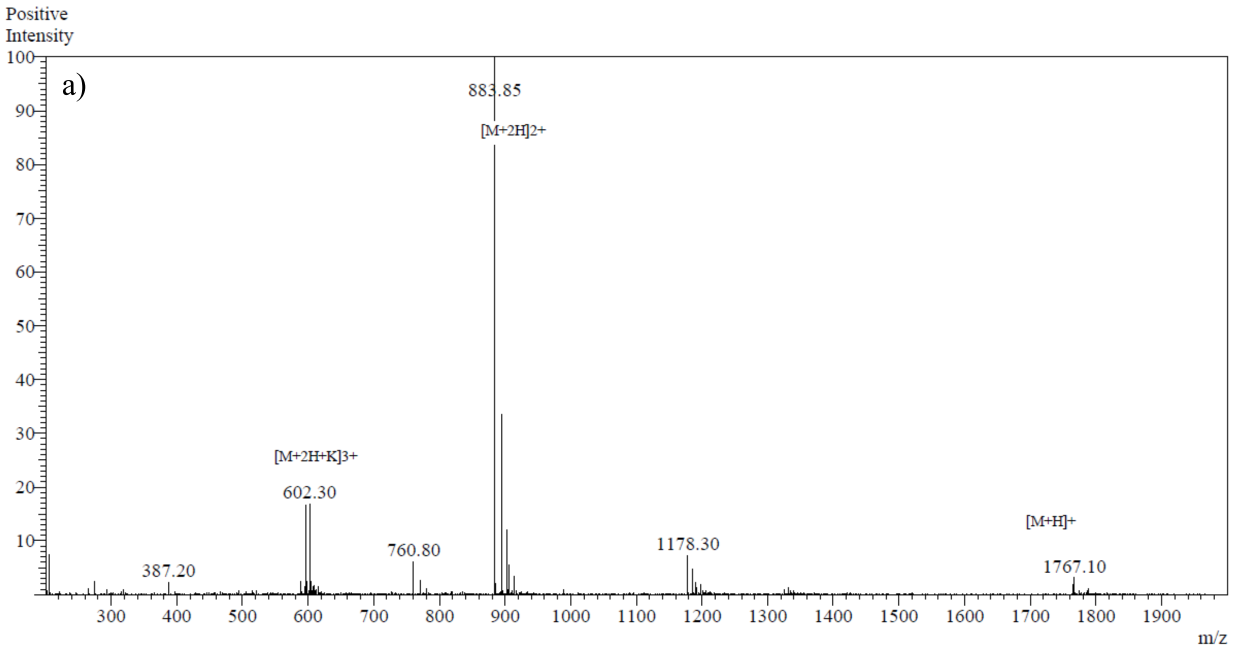

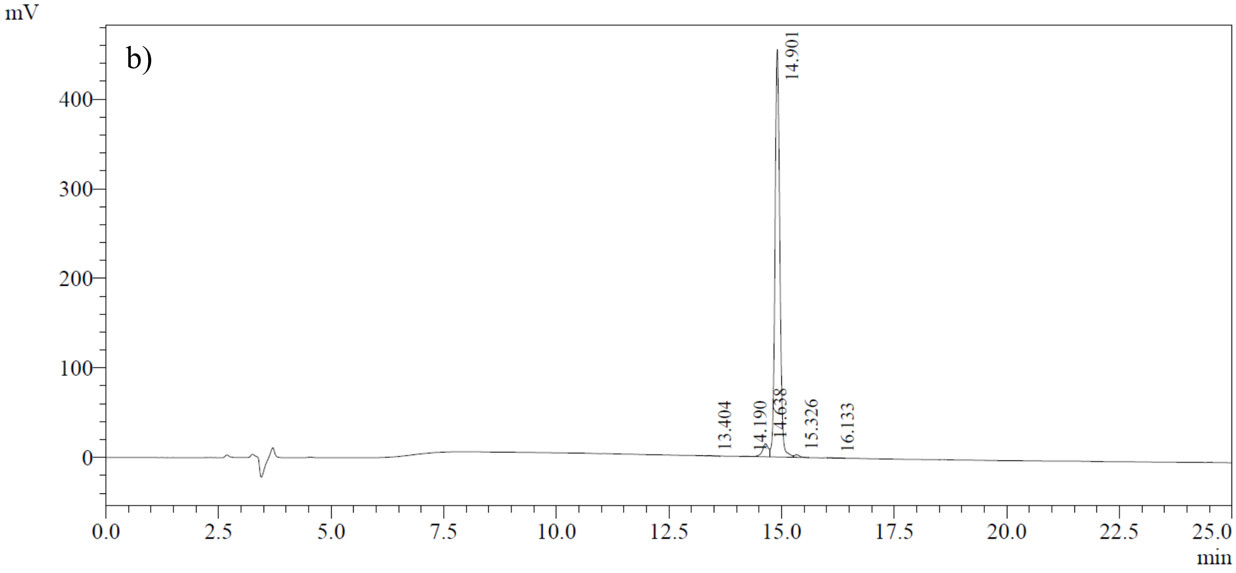

Supplementary Figure 5 Molecular weight and purity confirmation of pU24-6A peptide.

a) ESI spectra and b) chromatogram of purchased pU24-6A. The purity was determined to be 95.2%.

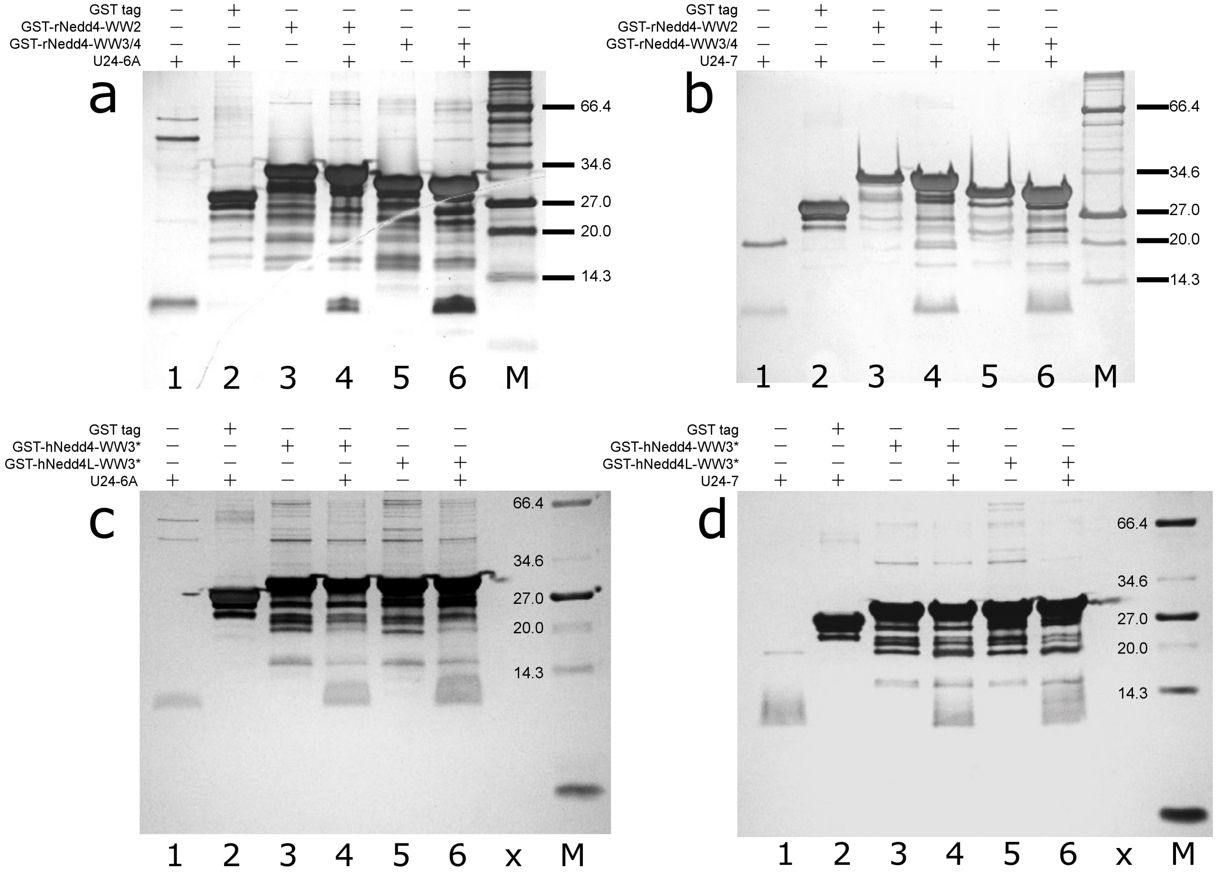

Supplementary Figure 6 Uncropped SDS-PAGE result of GST pull-down experiment with GST-Nedd4-WW domains and U24

Pull-down experiment using a) GST-rNedd4-WW2 and GST-rNedd4-WW3/4 and U24-6A protein, b) GST-rNedd4-WW2 and GST-rNedd4-WW3/4 and U24-7 protein, c) GST-hNedd4-WW3* and GST-hNedd4L-WW3* and U24-6A protein, and d) GST-hNedd4-WW3* and GST-hNedd4L-WW3* and U24-7 protein.

Supplementary Table 1 Thermodynamic parameters obtained from fitting ITC data for U24 peptides binding to Nedd4-WW domains at 25 °C in different buffers.

The numbers in the brackets are the error, i.e. ± one standard deviation from three runs. *n* indicates the number of binding sites.

extra NaCl concentration in 10 mM Na phosphate		U24-6A				U24-7			
		n (sites)	K_D (μM)	ΔH (kJ/mol)	ΔS (J/mol K)	*n* (sites)	K_D (μM)	ΔH (kJ/mol)	ΔS (J/mol K)
rNedd4-WW3/4	100 mM NaCl	1.169 (0.001)	35.7 (0.7)	-45.7 (0.5)	-68 (2)	1.04 (0.02)	14.6 (0.1)	-66 (3)	-129 (11)
	500 mM NaCl	1.191 (0.004)	30.6 (0.9)	-48.2 (0.7)	-75 (3)	1.117 (0.005)	13.2 (0.4)	-65.5 (0.6)	-126 (2)
hNedd4L-WW3*	100 mM NaCl	1.102 (0.005)	6.9 (0.2)	-58.3 (0.5)	-97 (2)	1.173 (0.002)	1.84 (0.06)	-69.4 (0.5)	-123 (2)
	500 mM NaCl	1.142 (0.004)	6.1 (0.2)	-57.4 (0.4)	-93 (2)	1.107 (0.002)	2.09 (0.04)	-73.1 (0.1)	-136.3 (0.6)

extra NaCl concentration in 10 mM Na phosphate		pU24-6A			
		n (sites)	K_D (μM)	ΔH (kJ/mol)	ΔS (J/mol K)
rNedd4-WW3/4	100 mM NaCl	1.046 (0.004)	12 (1)	-56 (2)	-93 (6)
	500 mM NaCl	1.03 (0.06)	9.5 (0.1)	-55.4 (0.7)	-90 (3)
hNedd4L-WW3*	100 mM NaCl	1.125 (0.004)	1.33 (0.03)	-61.1 (0.3)	-92 (1)
	500 mM NaCl	1.174 (0.006)	1.97 (0.03)	-53.8 (0.2)	-71.3 (0.9)

Supplementary Table 2 Total number of hydrophobic contacts and hydrogen bond interactions found in the MD simulations of hNedd4L-WW3* and U24-6A, U24-7, or Nogo-A (see Fig. 1a for sequences).

The interactions are counted if they are present for at least 60% of the simulation time.

hNedd4L-WW3* residue	U24-6A	U24-7	Nogo-A
H21[a]	4	1	0
K24	0	0	3
T25	0	0	1
T26	0	4	2
E29	0	11	0
R32	0	2	4
Total	4	18	10

[a] Corresponds to H498 in the actual numbering (**Fig. 1**).

Supplementary Table 3 Residues involved in the hydrophobic contacts (CT) and hydrogen bond (HB) interactions found in the MD simulations of hNedd4L-WW3* and U24-6A, U24-7, or Nogo-A.

hNedd4L-WW3*	U24-6A	U24-7	Nogo-A
H21[a]	CT: ND1 to OE2 in E13[b]<br>CT: ND1 to CD in E13<br>CT: ND1 to OE1 in E13<br>HB: HD1 to OE2 in E13	CT: NE2 to OH in Y10	N/A
K24	N/A	N/A	HB: O to H in Y11<br>CT: O to CA in P10<br>CT: O to N in Y11
T25	N/A	N/A	CT: CA to O in P9
T26	N/A	HB: OG1 to H in N11<br>CT: O to CD in P8<br>CT: OG1 to N in N11<br>CT: CG2 to CA in Y10	HB: H to O in P9<br>CT: N to O in P9
E29	N/A	CT: CD to OG1 in T5<br>CT: OE1 to OG1 in T5<br>CT: OE2 to N in T5<br>HB: OE2 to HG1 in T5<br>HB: OE1 to HG1 in T5<br>HB: OE2 to H in E4<br>CT: OE2 to N in E4<br>CT: OE1 to N in E4<br>CT: OE1 to N in T5<br>HB: OE1 to H in T5<br>HB: OE2 to H in T5	N/A
R32	N/A	CT: C to NE2 in H3<br>CT: O2 to NE3 in H3	CT: C to NE2 in H3<br>CT: O1 to NE2 in H3<br>HB: O1 to HE2 in H3<br>CT: O2 to NE2 in H3
Total	4	18	10

[a] Corresponds to H498 in the actual numbering (**Fig. 1**).

[b] The atoms names are as in the PDB. The interaction is listed as "atom name" for the residue in column 1 to the residue on the peptide.
